# Association of blood neutrophil-lymphocyte ratio with short-term prognosis and severity of tuberculosis meningitis patients without HIV infection

**DOI:** 10.1186/s12879-023-08438-y

**Published:** 2023-07-05

**Authors:** Zhihan Gu, Bofu Liu, Xiaomin Yu, Tao Cheng, Tianyong Han, Le Tong, Yu Cao

**Affiliations:** 1 Department of Emergency Medicine, Laboratory of Emergency Medicine, West China Hospital, West China School of Medicine, Sichuan University, Chengdu, China; 2 Disaster Medical Center, Sichuan University, Chengdu, Sichuan 610041 China

**Keywords:** Tuberculosis meningitis, Neutrophil-lymphocyte ratio (NLR), Prognosis, Severity

## Abstract

**Background:**

Predicting the short-term prognosis and severity of tuberculosis meningitis (TBM) patients without HIV infection can be challenging, and there have been no prior studies examining the neutrophil lymphocyte ratio (NLR) as a potential predictor of short-term prognosis or its relationship to TBM severity. We hypothesized that NLR might serve as an independent indicator of short-term prognostic significance and that there might be a correlation between NLR and severity. The aim of this study was to investigate the role of NLR as a predictor of short-term prognosis and its relationship to severity of tuberculosis meningitis patients without HIV infection.

**Methods:**

We retrospectively collected data from patients diagnosed with TBM in the West China Hospital, Sichuan University, from the period bet*w*een January 1st, 2018 and August 1st, 2019. Multivariable analysis was executed by the logistic regression model to verify the independence of the 28-day mortality, the discriminative power for predicting short-term prognosis was evaluated using a Receiver Operating Characteristic (ROC) curve, survival outcomes were analyzed using the Kaplan-Meier method and Pearson’s correlation analysis was performed to discuss correlation between NLR and the severity of TBM.

**Results:**

We collected data from 231 TBM patients without HIV infection. 68 (29.4%) patients are classified as stage (I) 138(59.8%) patients are stage (II) 25(10.8%) patients are stage (III) 16(6.9%) patients died during the follow-up period of 28 days. By multiple logistic regression analyses, the NLR (OR = 1.065, 95% CI = 1.001–1.133, P = 0.045), peripheral neurological deficit (OR 7.335, 95% CI 1.964–27.385, P = 0 0.003) and hydrocephalus (OR 11.338, 95% CI 2.397–53.633, P = 0 0.002) are independent risk factors of 28-day mortality. The area under the ROC curve (AUC) for predicting short prognosis using NLR is 0.683 (95% CI 0.540–0.826, P = 0.015), the optimal cutoff value is 9.99(sensitivity: 56.3%, specificity: 80.9%). The Kaplan-Meier analysis demonstrated that patients with higher NLR(>9.99) had significantly worse survival outcomes(P<0.01).Pearson’s correlation analysis presents a significant positive correlation between the severity of TBM and NLR (r = 0.234, P<0.01).

**Conclusions:**

NLR, peripheral neurological deficit, and hydrocephalus are independent risk factors of 28-day mortality, NLR can predict the short-term prognosis of TBM patients without HIV infection. NLR is also found to be significantly and positively correlated with the severity of TBM.

## Introduction

Tuberculosis (TB) is the major cause of death by an infectious disease worldwide [1]. However, tuberculosis meningitis (TBM) is considered the most devastating manifestation of Mycobacterium tuberculosis infection [2]. TBM is an inflammatory condition caused by the invasion of Mycobacterium tuberculosis into the human central nervous system, including the spinal cord, meninges, or brain parenchyma, which can lead to a series of secondary pathophysiological changes in the brain of affected patients [3]. The clinical course of TBM without HIV infection is nonspecific and quite variable. Additionally, various complex factors can impact the prognosis of patients with this disease. However, early identification of high-risk patients, along with intensive treatment, may help improve their prognosis [4]. So, assessment of the sufficient risk factor in patients with TBM is crucial in making clinical decisions.

Even given anti-tuberculosis treatment, The mortality rate of TBM patients as high as 10–50% [5]. Some studies indicated that factors such as disturbance of consciousness and hydrocephalus might be correlated with poor prognosis for patients with TBM [2, 5, 6]. However, the marker of inflammation in the blood has not be reported about the short-term prognosis of TBM patients.

Neutrophil-lymphocyte ratio (NLR) is defined as the number of neutrophils in whole blood divided by the number of lymphocytes in whole blood [7],it is a biomarker derived from leukocytes as a marker of inflammation. The NLR describes is a reliable parameter to describe the immune response to various stimuli/stressors [8] and it has been found to be a useful biomarker for predicting prognosis or severity in various clinical setting including sepsis, military tuberculosis and bacterial meningitis [8–10],but the association of NLR with clinical prognosis and severity in TBM patients without HIV infection has not been reported, This study is expected to determine whether NLR is an independent predictor of short-term prognosis in TBM patients without HIV infection and the relationship between NLR and the severity of TBM.

## Methods

### Patients and diagnosis of TBM

We retrospectively collected the data of all patients (≥ 18year-old) who were admitted to the West China Hospital, Sichuan University and diagnosed with TBM, from the period between January 1st, 2018 and August 1st, 2019. Patients were eligible for inclusion according to the standardized case definition of TBM proposed by Marais including the clinical criteria, CSF criteria, cerebral imaging criteria, evidence of tuberculosis elsewhere and exclusion of alternative diagnoses: An alternative diagnosis must be confirmed microbiologically, serologically, or histopathologically [11]. Patients with data was insufficient, HIV infection and pregnant were excluded from the study.

### Collection of clinical data

The data information of patients was obtained by the patients’ electronic medical record management system and telephone. At the time of admission, the following data were collected, including demographic information (sex, age, disease duration), clinical criteria (fever (> 37.5℃) > 5 days, night sweats, weight loss, cough, headache, vomiting, peripheral neurological deficit, cognitive impairment, change in consciousness, cranial nerve palsy, and seizures), laboratory data (red blood cell, white blood cell, platelet, neutrophil, lymphocyte, the neutrophil- lymphocyte ratio(NLR), Serum sodium, Serum potassium, plasma glucose, albumin, CSF leukocyte count, CSF protein, CSF chlorine, CSF glucose, ratio of CSF to blood glucose), and cerebral imaging criteria (hydrocephalus, basal meningeal enhancement, tuberculoma, and infarct).We collected the severity of TBM at the time of admission which was assessed using the British Medical Research Council (BMRC) TBM stages modified as grade I (GCS 15; no focal neurological signs), grade II (GCS 11–14, or 15 with focal neurological signs), and grade III (GCS ≤ 10)disease [11] ,and also collected the outcome (survivors and non- survivors)after 28 day via telephone call.

### Management

Patients received four TBM treatments, isoniazid (10–20 mg/kg/day; maximum 1200 mg/day), rifampicin (10–20 mg/kg/day; maximum 600 mg/day), pyrazinamide (20–30 mg/kg/day; maximum 1500 mg/ day), and ethambutol: (15–20 mg/kg/day; maximum 750 mg/day), they also received dexamethasone(0.4 mg/kg/day; maximum16mg/day) at the same time of diagnosis, administered for 4 weeks.

### Statistical analysis

A chi-squared or Fisher’s exact test was implemented to compare the categorical variables. An independent-sample test was utilized to compare the continuous variables. Multivariable analysis was executed by the stepwise logistic regression model (forward) to verify the independence of the 28-day mortality, and the Lemeshow-Hosmer χ2 statistics was used to evaluate the Goodness-of-fit of the Multivariable logistic regression model. The discriminative power for predicting short-term prognosis was evaluated using a Receiver Operating Characteristic (ROC) curve, survival outcomes were analyzed using the Kaplan-Meier method. Pearson’s correlation analysis was performed to discuss correlation about and the severity of TBM. All statistical analyses were implemented with the use of SPSS software version 22.0 (USA, IBM analytics). A P value equal or less than 0.05 was statistically significant in all analyses.

## Results

There were 245 patients with TBM who were treated at the West China Hospital, Sichuan University. 14 patients were excluded from the study because of insufficient data or HIV infection, finally, a total of 231 patients were analyzed in this study. During the study period, 16 patients (6.9%) died while the remaining 215 (93.1%) survived.

In the study, there are 129 (55.8%) men, the average age of all patients is 36 ± 16years. The patients are classified into three stages according to the severity: 68 (29.4%) are in stage I, 138 (59.8%) are in stage II, and 25 (10.8%) are in stage III.

Tables 1 and 2 describes the patients’ characteristics between the survivors and deaths. The most common symptoms of TBM are fever, headache, and vomiting, with an incidence rate of over 50%. Survivors exhibit a lower incidence of peripheral neurological deficit (P < 0.001), change in consciousness (P = 0.002), and hydrocephalus (P < 0.001) compared to those who died. NLR are higher in the deaths compared with survivors (P = 0.003), and albumin are lower in the deaths (P = 0.045). There is no significant statistical difference in the other factors.

**Table 1:** The TBM^1^ patient’s general characteristics without HIV infection

Variable	Overall cases (n = 231)	Survivors (n = 215)	Non-survivors (n = 16)	P
Sex				
Male, n, %	129/55.8%	119(55.3%)	10(62.5%)	0.578
Age, years, mean ± sd	36 ± 16	36 ± 16	34 ± 16	0.618
Disease duration^a^, day, median,Interquartile range 25%,75%)	28(14, 60)	28(14, 60)	25(12, 32)	0.559
Fever(> 37.5℃) > 5days, n,(%)	188(81.4%)	177(82.3%)	11(68.8%)	0.178
Night sweats, n, (%)	53(22.9%)	51(23.7%)	2(12.5%)	0.303
Weight loss n, (%)	80(34.0%)	76(34.9%)	4(25%)	0.587
Cough, n, (%)	50(21.6%)	45(20.9%)	5(31.3%)	0.334
Headache, n, (%)	210(90.9%)	197(91.6%)	13(81.3%)	0.164
Vomiting, n, (%)	150(63.8%)	141(64.4%)	9(56.3%)	0.513
Peripheral neurological deficit^b^, n, (%)	69(29.9%)	57(26.5%)	12(75.0%)	0.000
Cognitive impairment, n, (%)	42(18.2%)	37(17.2%)	5(31.3%)	0.160
Change in consciousness, n, (%)	103(44.6%)	90(41.9%)	13(81.3%)	0.002
Cranial nerve palsy, n, (%)	57(24.7%)	51(23.7%)	6(37.5%)	0.217
Seizures, n, (%)	39(16.9%)	34(15.8%)	5(31.3%)	0.112

***Note***: ^1^TBM: Tuberculosis meningitis; ^a^Disease duration: Length of time from onset to hospital treatment; ^b^Peripheral neurological deficit: the patient has limb numbness, limb movement disorder, fine motor loss and other manifestations

**Table 2:** The TBM^1^ patient’s laboratory and imaging characteristics without HIV infection

Variable	Overall cases (n = 231)	Survivors (n = 215)	Non-survivors (n = 16)	P
Laboratory data				
Red blood cell,10^12/L, mean ± sd	4.44 ± 0.59	4.44 ± 0.59	4.51 ± 0.65	0.651
Platelet 10^9/L, mean ± sd	240 ± 100	241 ± 102	225 ± 67	0.546
White blood cell,10^9/L, mean ± sd	7.95 ± 3.46	7.84 ± 3.25	9.54 ± 5.53	0.244
Neutrophil, 10^9/L, mean ± sd	6.19 ± 3.37	6.06 ± 3.19	7.86 ± 5.08	0.181
Lymphocyte, 10^9/L, mean ± sd	1.13 ± 0.71	1.15 ± 0.72	0.87 ± 0.64	0.132
The neutrophil- lymphocyte ratio(NLR),%	7.58 ± 6.66	7.21 ± 6.31	12.51 ± 9.22	0.003
Serum sodium, mmol/L, mean ± sd	132.1 ± 7.7	131.9 ± 7.8	135.0 ± 5.5	0.122
Serum potassium, mmol/L, mean ± sd	3.70 ± 0.47	3.71 ± 0.47	3.56 ± 0.48	0.212
Plasma glucose, mmol/L, mean ± sd	6.09 ± 1.47	6.07 ± 1.48	6.41 ± 1.22	0.374
Albumin, g/L, mean ± sd	38.4 ± 5.0	38.5 ± 4.9	35.9 ± 6.5	0.045
CSF^a^ leukocyte count, cell/ul,median,Interquartile range 25%,75%)	120(60,270)	120(60, 270)	125(43, 350)	0.932
CSF^a^ protein, g/L, median,Interquartile range 25%,75%)	1.67(1.09,2.56)	1.56(1.08, 2.54)	2.29(1.17, 4.90)	0.104
CSF^a^ chlorine, mmol/L, mean ± sd	113.93 ± 8.37	114.10 ± 8.27	111.83 ± 9.74	0.299
CSF^a^ glucose, mmol/L, mean ± sd	2.05 ± 1.02	2.05 ± 0.99	2.06 ± 1.40	0.952
Ratio of CSF^a^ to blood glucose, mean ± sd	0.353 ± 0.186	0.354 ± 0.182	0.336 ± 0.234	0.697
Cerebral imaging criteria				
Hydrocephalus, n, (%)	73(31.6%)	59(27.4%)	14(87.5%)	0.000
Infarct, n, (%)	94(40.7%)	85(39.5%)	9(56.3%)	0.189
Basal meningeal enhancement, n, (%)	78(33.8%)	71(33.0%)	7(43.8%)	0.381
Tuberculoma, n, (%)	7(3.0%)	6(2.8%)	1(6.3%)	0.399

***Note***: ^1^TBM: Tuberculosis meningitis; ^a^CSF: Cerebrospinal fluid

In this study, seven single factors including peripheral neurological deficit, change in consciousness, hydrocephalus, NLR, albumin, age, and seizures were included in a multiple logistic regression analysis, and it was found that the NLR is an independent risk factor for 28-day mortality (OR = 1.065, 95% CI = 1.001–1.133, P = 0.045). In addition, peripheral neurological deficit (OR 7.335, 95% CI 1.964–27.385, P = 0.003) and hydrocephalus (OR 11.338, 95% CI 2.397–53.633, P = 0 0.002) were significantly more likely in deceased patients than in survivors (refer to Table 3). Neither the rate of change in consciousness, albumin, age and seizures are independent risk factors for 28-day mortality of patients with TBM. The Hosmer-Lemeshowχ2 statistics of the multivariate logistic regression models is 2.260 (P = 0.972).

**Table 3:** The multivariate analysis result ^1^of 28-day mortality in TBM^2^ patients without HIV infection

Variable	OR^b^	95%CI^c^	P
The neutrophil lymphocyte ratio(NLR)	1.065	1.001, 1.133	0.045
Peripheral neurological deficit^a^	7.335	1.964, 27.385	0.003
Hydrocephalus	11.338	2.397, 53.633	0.002

***Note***: ^1^The multivariate analysis result was adjusted to age, clinical presentations, and albumin levels; ^2^ TBM: Tuberculosis meningitis; ^a^Peripheral neurological deficit: the patient has limb numbness, limb movement disorder, fine motor loss and other manifestations; ^b^OR: odds ratio; ^c^CI: confidence interval

**Fig. 1:**
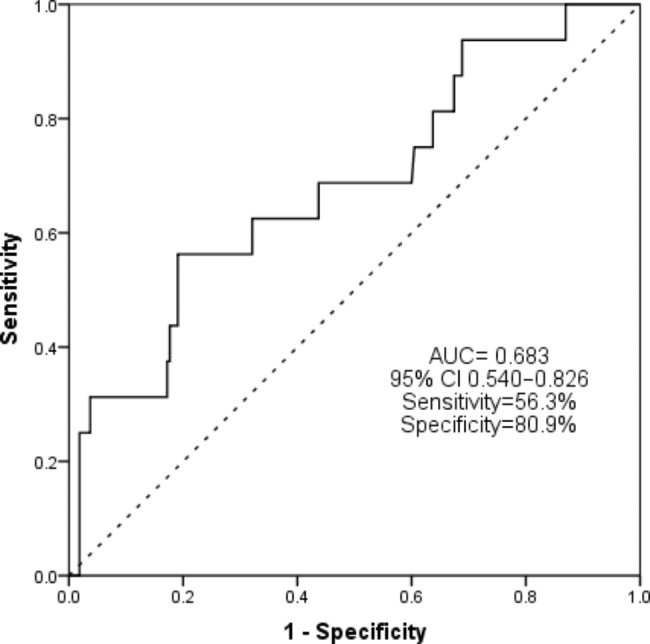
The ROC curves for predicting short-term prognosis in TBM patients without HIV infection by using NLR

The area under the ROC curve (AUC) for predicting short-term prognosis in patients with TBM using NLR is 0.683 (95% CI 0.540–0.826, P = 0.015), with an optimal cutoff value of 9.99. When using this cutoff, NLR has a sensitivity of 56.3% and specificity of 80.9% for predicting short-term prognosis (refer to Fig. 1).

**Fig. 2:**
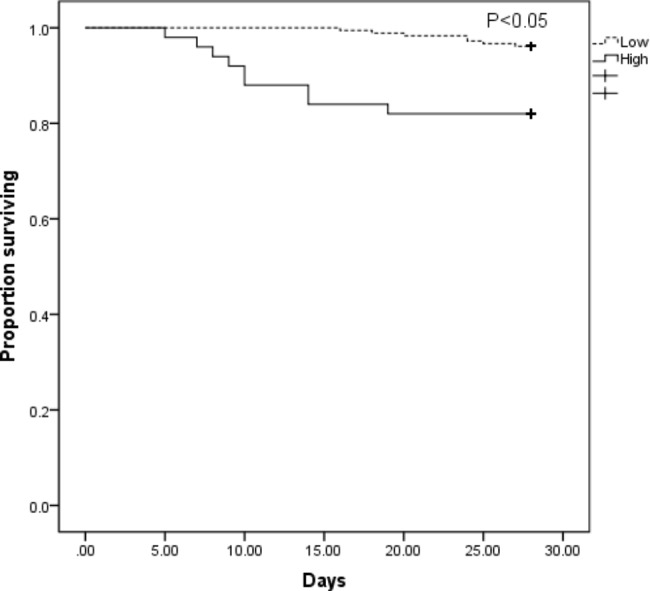
The results of the Kaplan-Meier analysis that TBM patients with high NLR and low NLR

Patients were classified into the high NLR group and the low NLR group using a cutoff value of 9.99. Compared to the low NLR group, patients in the high NLR group exhibited higher incidence rates of changes in consciousness, hydrocephalus, and basal meningeal enhancement. Furthermore, they had significantly elevated levels of white blood cells and plasma glucose, and significantly reduced levels of albumin, serum sodium, CSF glucose, CSF chlorine, and the ratio of CSF to blood glucose (refer to Tables 4 and 5). Moreover, patients in the high NLR group had a higher 28-day mortality rate (refer to Table 5). The Kaplan-Meier analysis demonstrated that patients with high NLR had significantly worse survival outcomes (P < 0.05) (refer to Fig. 2).

**Table 4:** Comparison of general characteristics between patients with high and low NLR^1^

Variable	Overall cases (n = 231)	Survivors (n = 215)	Non-survivors (n = 16)	P
Sex				0.729
Male, n, %	129/55.8%	100(55.2%)	29(58.0%)	
Age, years, mean ± sd	36 ± 16	36 ± 16	37 ± 18	0.561
Fever(> 37.5℃) > 5days, n,(%)	188(81.4%)	148(81.8%)	40(80%)	0.776
Night sweats, n, (%)	53(22.9%)	43(23.8%)	10(20.0%)	0.576
Weight loss n, (%)	80(34.0%)	64(35.3%)	16(32%)	0.693
Cough, n, (%)	50(21.6%)	37(20.4%)	13(26.0%)	0.398
Headache, n, (%)	210(90.9%)	167(92.3%)	43(86%)	0.173
Vomiting, n, (%)	150(63.8%)	141(64.4%)	9(56.3%)	0.513
Peripheral neurological deficit^a^, n, (%)	69(29.9%)	52(28.7%)	17(34%)	0.471
Cognitive impairment, n, (%)	42(18.2%)	32(17.7%)	10(20.0%)	0.706
Change in consciousness, n, (%)	103(44.6%)	70(38.7%)	33(66.0%)	0.001
Cranial nerve palsy, n, (%)	57(24.7%)	44(24.3%)	13(26.0%)	0.806
Seizures, n, (%)	39(16.9%)	30(16.6%)	9(18.0%)	0.812

***Note***: ^1^ NLR: neutrophil-lymphocyte ratio; ^a^Peripheral neurological deficit: the patient has limb numbness, limb movement disorder, fine motor loss and other manifestations

**Table 5:** Comparison of laboratory characteristics, imaging characteristics and prognosis between patients with high NLR^1^ and low NLR^1^

Variable	Overall cases (n = 231)	Low (n = 181)	High (n = 50)	P
Laboratory data				
Red blood cell,10^12/L, mean ± sd	4.44 ± 0.59	4.47 ± 0.58	4.36 ± 0.63	0.270
Platelet 10^9/L, mean ± sd	240 ± 100	237 ± 95	249 ± 116	0.472
White blood cell,10^9/L, mean ± sd	7.95 ± 3.46	7.23 ± 2.90	10.56 ± 4.08	0.000
Serum sodium, mmol/L, mean ± sd	132.1 ± 7.7	132.9 ± 7.6	129.4 ± 7.4	0.004
Serum potassium, mmol/L, mean ± sd	3.70 ± 0.47	3.72 ± 0.46	3.63 ± 0.49	0.250
Plasma glucose, mmol/L, mean ± sd	6.09 ± 1.47	5.91 ± 1.42	6.76 ± 1.47	0.000
Albumin, g/L, mean ± sd	38.4 ± 5.0	38.9 ± 4.5	36.4 ± 6.2	0.002
CSF^a^ leukocyte count, cell/ul,median,Interquartile range 25%,75%)	120(60,270)	110(60, 275)	130(58, 253)	0.933
CSF^a^ protein, g/L, median,Interquartile range 25%,75%)	1.67(1.09,2.56)	1.67(1.04, 2.54)	1.62(1.17, 2.79)	0.456
CSF^a^ chlorine, mmol/L, mean ± sd	113.93 ± 8.37	115.12 ± 8.04	109.64 ± 8.20	0.000
CSF^a^ glucose, mmol/L, mean ± sd	2.05 ± 1.02	2.17 ± 1.04	1.58 ± 0.81	0.000
Ratio of CSF^a^ to blood glucose, mean ± sd	0.353 ± 0.186	0.383 ± 0.185	0.247 ± 0.147	0.000
Cerebral imaging criteria				
Hydrocephalus, n, (%)	73(31.6%)	46(25.4%)	27(54.0%)	0.000
Infarct, n, (%)	94(40.7%)	71(39.2%)	23(46.0%)	0.388
Basal meningeal enhancement, n, (%)	78(33.8%)	55(30.4%)	23(46.0%)	0.039
Tuberculoma, n, (%)	7(3.0%)	5(2.8%)	2(4.0%)	0.647
28-day mortality n, (%)	16(6.9%)	7(3.9%)	9(18.0%)	0.000

***Note***: ^1^ NLR: neutrophil-lymphocyte ratio; ^a^CSF: Cerebrospinal fluid

The Study also explored the relationship between the severity of TBM and the NLR. The NLR values for patients with stage I TBM are 6.00 ± 5.03, while those for patients with stage II TBM are 7.56 ± 6.33, and for patients with stage III TBM, the NLR values are 12.00 ± 9.92. Pearson’s correlation analysis presents a significant positive correlation between the severity of TBM and NLR (r = 0.234, P < 0.01).

## Discussion

In this study, NLR was found to be an independent predictor of short-term prognosis in TBM patients without HIV infection. Patients with NLR greater than 9.99 had worse survival outcomes. In a number of studies, it has been reported that high NLR is associated with high mortality in cancers [12–14] or cardiovascular diseases [9, 15, 16]. NLR is a readily obtainable inflammatory marker that indicates the degree of neutrophil elevation or lymphocyte depletion. At present, the cause for the correlation between the increase in NLR and poor outcome has not yet been explained clearly. High neutrophil counts or lymphopenia may be associated with poor prognosis in several previous studies on TB [17–19], but NLR has not been represented in TBM patients. Chedid, C hypothesized that high neutrophil counts and low lymphocyte proportions had highly inflammatory clinical patterns by the context of mycobacterial infection [17], which are markers of persisting inflammation or failure to clear the bacteria [20]. High neutrophil counts may reflect the presence of delayed apoptosis in inflammatory condition, and inhibit the anti-inflammatory ability of neutrophil cells [21]. In chronic TB infection, sustained inflammation which was an impairment maker of the TB-specific immune response and a marker of active disease has be reported [22]. High NLR may represent relative lymphopenia, low lymphocyte counts are associated with a negative response to treatment [17, 23]in relation to cell-mediated immunity which is important in the immune response to M. tuberculosis [9]. So, the similar pathway may have affected the progression and prognosis of TBM.

The study also investigates the association of high NLR with the severity of TBM. The higher NLR represents the higher the clinical stage, as some studies have reported a link between high NLR and higher cancer stage [13, 19, 24] and the severity of pulmonary TB [25]. When the NLR of TBM patients is greater than 9.99, they are more likely to experience altered consciousness and develop complications such as hydrocephalus, hyponatremia, and hypoproteinemia. These factors indicate that the patient may have a higher severity.

In this study, peripheral neurological deficit is a predictor of poor prognosis in TBM patients. A series of studies had reported similar conclusion [26–28]. About 15–50% of patients with TBM have exist peripheral neurological deficit when they first present symptom [27–30], this study reported 30% (69/231) of patients with TBM. This condition may be associated with high rates of morbidity and mortality. Some studies also reported cranial nerve palsy is a risk factors association with bad prognosis [30, 31],but this study did not support it. This study focused the 28-day mortality however those studies had a long-term follow-up result.

Hydrocephalus is a quite common complication of TBM, as reported, up to 60% of patients with TBM be found to by cranial imaging such as MRI/CT [32]. Patients with hydrocephalus may be associated with the pathogenesis of meningeal exudate, as well as either overproduction of cerebrospinal fluid (CSF) or malfunctioning absorption of CSF in the subarachnoid space [33, 34], and it is considered to be an important risk factor for poor prognosis.

### Limitations

This study suggests that NLR is a useful predictor of short-term prognosis for TBM patients without HIV infection, but it has several limitations. Firstly, the sample size was small, with only 16 deaths occurring within 28 days, which could potentially affect the stability of the logistic model. Additionally, the diagnosis primarily relied on clinical criteria, CSF criteria, and cerebral imaging criteria, which may introduce bias into the data. This study did not exclude patients who temporarily discontinued anti-tuberculosis treatment due to medication side effects. Furthermore, potential drug-resistant patients were not identified due to the lack of drug resistance testing. These factors have the potential to impact the study’s results. Further studies with larger sample sizes are necessary to validate the effect of NLR values on the short-term prognosis of TBM patients without HIV infection.

## Conclusions

NLR, peripheral neurological deficit, and hydrocephalus are independent risk factors of 28-day mortality, NLR can predict the short-term prognosis in TBM patients without HIV infection. NLR is also found to be significantly and positively correlated with the severity of TBM. Therefore, TBM patients without HIV infection who have high NLR values should be closely monitored in clinical practice.

## Data Availability

All data generated or analyzed during this study are included in this published article.

## Abbreviations

BMRC: British Medical Research Council
CI: Confidence interval
CNS: Central nervous system
CSF: Cerebrospinal fluid
GCS: Glasgow Coma Scale
HIV: Human immunodeficiency virus
NLR: Neutrophil-lymphocyte ratio
OR: Odds ratio
ROC: Receiver Operating Characteristic
TB: Tuberculosis
TBM: Tuberculosis meningitis
